# No evolutionary change in the mating system of *Solanum rostratum* (Solanaceae) during its invasion in China

**DOI:** 10.1038/s41598-017-17881-2

**Published:** 2017-12-18

**Authors:** Lijuan Zhang, Ling Yu, Anru Lou

**Affiliations:** 1 State Key Laboratory of Earth Surface Processes and Resource Ecology, Beijing Normal University, Beijing, 100875 China; 2 State Key Laboratory of Earth Surface Processes and Resource Ecology, College of life Sciences, Beijing Normal University, Beijing, 100875 China

## Abstract

The mating system of flowering plants plays a key role during the process of invasion. Evolution from outcrossing to selfing can allow rapid regeneration of a population after long-distance dispersal by providing reproductive assurance. *Solanum rostratum* is a self-compatible annual herb that exhibits a high level of outcrossing in its native populations. However, the mating system of invasive populations of *S. rostratum* has never been assessed. Here, we investigated the mating system based on 11 microsatellite loci and explored ecological factors that may influence the outcrossing rate among 10 invasive populations of *S. rostratum* in China. We found that the mean outcrossing rate was 0.69 ± 0.12 (range 0.49 to 0.83) with multiple paternity within progeny arrays (average effective number of sires = 7.86), which suggests a mixed mating system dominated by outcrossing. Combined with the uniformly high outcrossing rate (0.70 ± 0.03) previously reported in its native range, these results indicate that there has been no evolutionary shift in mating system during the invasion in China by *S. rostratum*. There were no relationships between outcrossing and population size, population density, altitude, latitude or longitude. Furthermore, high outcrossing of *S. rostratum* in China may be facilitated by enantiostyly and heteranthery.

## Introduction

The mating system of plant is the pattern in which gametes combine in plant populations^1^, and can influence the frequency of genes within the population^1,2^. As a result, the mating system of invasive plant species plays a fundamental role in determining the genetic diversity and genetic structure of a population and is closely related to adaptive evolution and invasion^1,2^. When alien flowering species are introduced to a new region, these species often occur at low density and experience increased pollen limitation because of the reduction of potential mates and suitable pollinators^3–5^. Such negative conditions can persist for a long time after the initial founder event resulting in Allee effects^6,7^, which can limit the spread of invaders. Therefore, natural selection may favor an association between selfing and colonizing ability^1,8^, as selfing can allow a rapid build-up of a population after long-distance dispersal by providing reproductive assurance according to Baker’s Law^9–11^. Efforts to explore the mating system of invasive species constitute an important task to better understand the biology of plant invasions and to predict microevolutionary changes in various environments.

Recent studies have used molecular techniques to evaluate selection on mating systems during biological invasion by comparing outcrossing rates between invasive and natural populations^1^. The evolutionary transition from outcrossing to selfing has occurred in some invasive plants, such as *Eichhornia paniculata and Eichhornia crassipes* ^12–15^, owing to the stochastic loss of mating types in these species. There are also some cases that have no evolutionary shift in the mating system during invasion, such as those involving *Ambrosia artemisiifolia* and *Senecio inaequidens* ^11,16^, owing to self-incompatibility. However, the evolution of mixed mating strategies, a mixture of self-fertilization and cross-fertilization, may be more complex^2,17^. And studies on the maintenance of mixed mating systems in hermaphroditic invasive plants have only been conducted relatively recently.

For animal-pollinated plants, there are evidences that the mating system of them is influenced not only by biotic factors, such as floral display, pollinator type and population size^17–19^, but also by environmental conditions, including geographic location and habitat fragmentation^20,21^. However, to the best of our knowledge, only a handful of studies^18^ have assessed the impact of biotic factors and environmental conditions on mating patterns of invasive species. Thus, it is necessary to investigate the factors that influence the mating system of invasive plants.

*Solanum rostratum* (Solanaceae) is a buzz-pollinated, self-compatible annual herb with hermaphroditic flowers. The species is believed to be native to Mexico and the USA^22^, but it has spread to China, Europe, Russia, Canada, South Korea, and Australia^22–24^. *S. rostratum* was found for the first time in Liaoning province in 1981, and has spread across a large area in North China during the last 36 years^24^. Populations of *S. rostratum* occur in disturbed habitats such as roadsides, riversides, sides of railways and abandoned fields in China, which is as same as the habitats in its origin area. The main pollinators of *S. rostratum* are bees in the genera *Bombus* and *Xylocopa* in both invasive and native populations. The bright yellow flowers of *S. rostratum* in China exhibit monomorphic enantiostyly and strong herkogamy^25,26^ as in its native region. The species has two mirror-image floral morphs alternating along the inflorescence, with flowers that present style and pollinating anther opposite each other, situated either right or left of the floral axis. The stamens of *S. rostratum* are divided into four small yellow feeding stamens and a single tinged brown large pollinating stamen according to their different functions^27^. The four centrally located feeding stamens serve to attract and reward bees, and the single pollinating stamen, which is deflected to either the right- or left-hand side of the flower opposite the style, contributes disproportionately to pollen reaching the stigmas of other flowers^27^. These special floral traits promote outcrossing by reducing selfing between the same flower morph^28,29^; in its native Mexican range, *S. rostratum* is highly outcrossed^30^. However, the mating system of invasive populations of *S. rostratum* has never been assessed.

Here, we investigated the mating system of *S. rostratum* among ten invasive populations in China and the effect of population size and other ecological factors on mating in *S. rostratum*. The present study had three objectives concerning the reproductive and invasive biology of *S. rostratum*. First, we assessed the mating system of *S. rostratum* by quantifying its mating system parameters using microsatellite markers. Second, we compared the outcrossing rate of Chinese populations with that of native populations in Mexico. Finally, we evaluated relationships between outcrossing rates and population size and other ecological factors such as altitude and latitude. Our study provides the first detailed evidence of the mating system of *S. rostratum* in its invasive Asian range.

## Results

### Mating system

**Figure 1:**
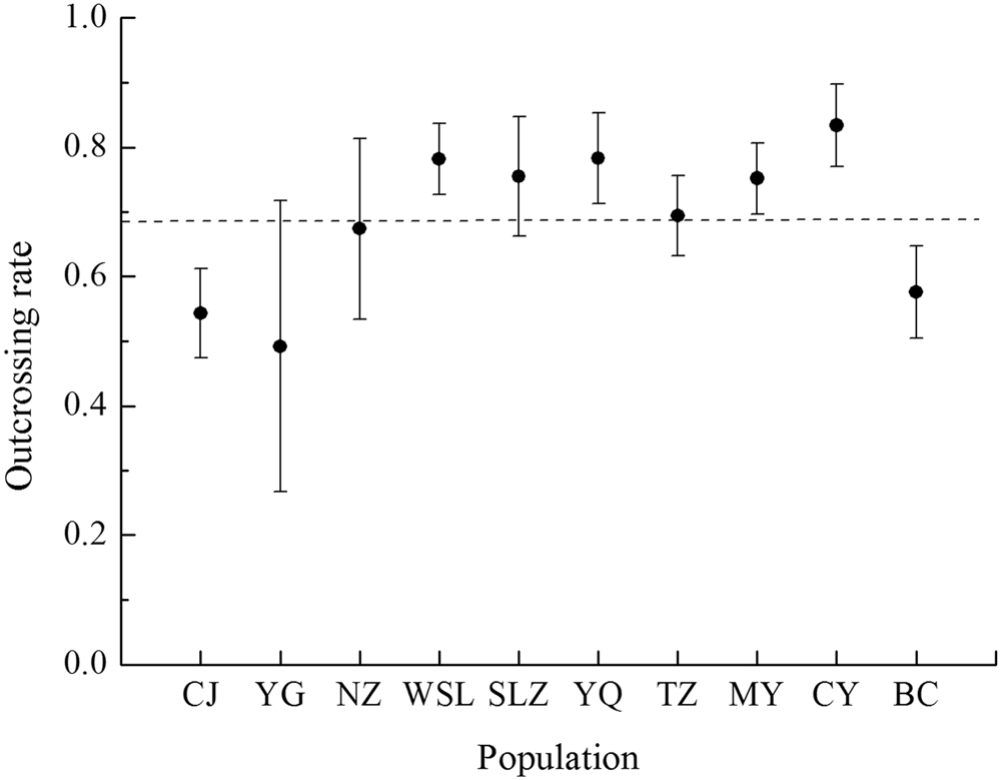
Multilocus outcrossing rates (*t*_*m*_) with 95% confidence intervals of 1000 bootstrap estimates from ten Chinese populations of *Solanum rostratum*. The dashed line shows the average outcrossing rate across all populations (${\overline{t}}_{{\rm{m}}}$ = 0.69 ± 0.12). Populations are listed according to the longitude. Population names as those in Table 1.

**Figure 2:**
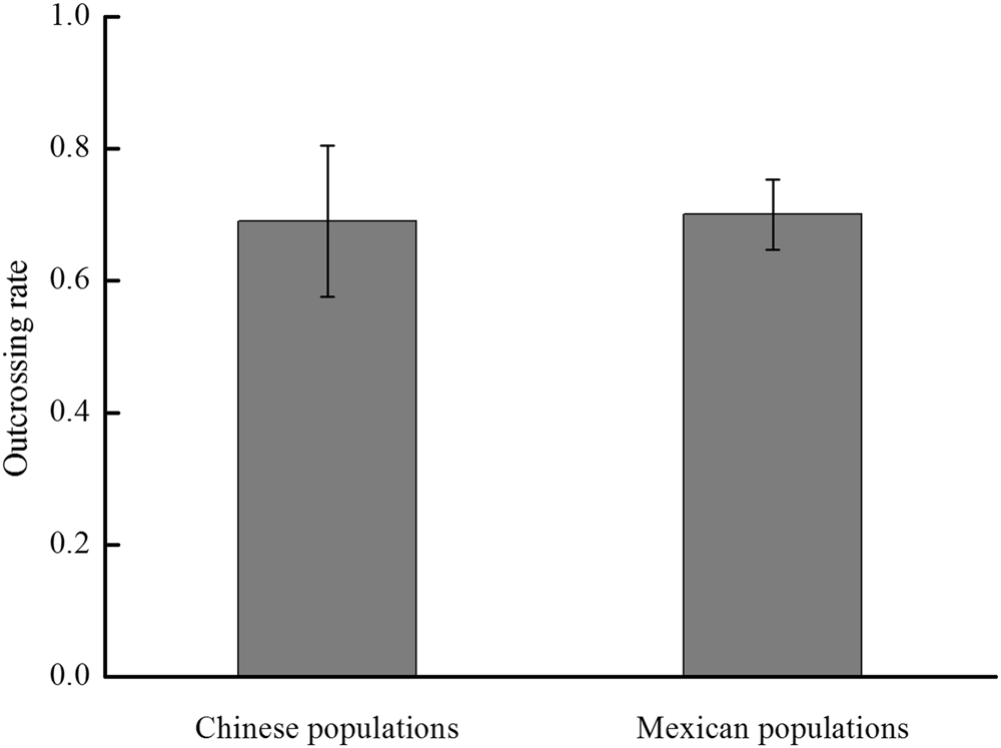
The average multilocus outcrossing rate (*t*_*m*_) across Chinese populations and Mexican populations of *Solanum rostratum*. The *t*_*m*_ of Mexican populations was reported by Vallejo-Marίn^30^.

The invasive populations of *S. rostratum* in China showed intermediate to high outcrossing rates (Table 1; Fig. 1). The average multilocus outcrossing rate (*t*_*m*_) across Chinese populations was 0.69 ± 0.12 (mean ± SD), ranging from 0.492 ± 0.225 in population YG to 0.834 ± 0.064 in population CY. The independent samples t-test showed that the outcrossing rates of Chinese populations had no significant difference with the previously reported outcrossing rates of Mexican populations^30^ (*P* > 0.05; Fig. 2). These results indicate that there has been no evolutionary shift in the mating system during the invasion of China by *S. rostratum*.

**Table 1:** Mating system parameters in ten chinese populations of *Solanum rostratum*.

Code	Population	*t*_m_	*t*_s_	*t*_m_ − *t*_s_	*r*_t_	*r*_pm_	*r*_ps_	*r*_ps_ − *r*_pm_
BC	Baicheng, Jilin	0.576^**^ (0.071)	0.567^**^(0.058)	0.009 (0.027)	0.103 (0.092)	−0.033 (0.072)	−0.152 (0.135)	−0.119 (0.119)
CJ	Changji, Xinjiang	0.543^**^ (0.069)	0.513^**^(0.060)	0.030 (0.026)	0.124 (0.068)	0.262 (0.141)	0.205 (0.180)	−0.057 (0.070)
CY	Chaoyang, Liaoning	0.834^**^ (0.064)	0.780^**^(0.070)	0.054 (0.031)	0.235 (0.211)	0.045 (0.029)	0.029 (0.031)	−0.016 (0.027)
YG	Yanggao, Shanxi	0.492^**^ (0.225)	0.451^**^(0.212)	0.041 (0.022)	0.505 (0.379)	0.046 (0.272)	−0.009 (0.331)	−0.055 (0.149)
WSL	Zhangjiakou, Hebei	0.782^**^ (0.055)	0.644^**^(0.058)	0.137 (0.023)	0.121 (0.095)	0.082 (0.022)	0.036 (0.039)	−0.046 (0.031)
NZ	Zhangjiakou, Hebei	0.674^**^ (0.140)	0.676^**^(0.147)	−0.002 (0.050)	0.318 (0.143)	0.227 (0.082)	0.142 (0.101)	−0.085 (0.079)
SLZ	Zhangjiakou, Hebei	0.775^**^ (0.092)	0.724^**^(0.088)	0.051 (0.035)	0.385 (0.196)	0.124 (0.042)	0.074 (0.083)	−0.050 (0.059)
MY	Miyun, Beijing	0.752^**^ (0.055)	0.705^**^(0.057)	0.047 (0.026)	0.039 (0.074)	0.213 (0.064)	0.176 (0.084)	−0.036 (0.033)
YQ	Yanqing, Beijing	0.783^**^ (0.070)	0.671^**^(0.079)	0.112 (0.029)	0.142 (0.112)	0.124 (0.055)	0.094 (0.063)	−0.030 (0.035)
TZ	Tongzhou, Beijing	0.694^**^ (0.062)	0.617^**^(0.059)	0.077 (0.045)	0.161 (0.079)	0.183 (0.043)	0.185 (0.056)	0.002 (0.036)

Note: SDs are presented in parentheses. *t*_m_, multilocus outcrossing rate; *t*_s_, single-locus outcrossing rate; *t*_m_ − *t*_s_, outcrossing rate between related individuals; *r*_t_, correlation of outcrossing rate; *r*_ps_, correlation of paternity within sibships for single cases; *r*_pm_, correlation of paternity within sibships for multilocus cases; *r*_ps_ − *r*_pm_, extent of outcrossed paternity by related male parents. ^**^ *P*-value < 0.01.

Estimates for the outcrossing rate between related individuals (*t*_m_−*t*_s_) were significantly greater than zero (range 0.009 ± 0.027 to 0.137 ± 0.023), except for population NZ (−0.002 ± 0.05), suggesting some degree of biparental inbreeding in most Chinese populations.

Estimates for the outcrossing rate between related individuals (*t*_m_−*t*_s_) were significantly greater than zero in both Chinese populations (range 0.009 ± 0.027 to 0.137 ± 0.023, except for population NZ −0.002 ± 0.05) and Mexican populations (range 0.034 ± 0.024 to 0.072 ± 0.028)^30^, suggesting some degree of biparental inbreeding in most populations. The correlation of paternity within sibships for multilocus cases (*r*_pm_) was low in all populations (0.13 ± 0.09), suggesting that approximately one in ten maternal siblings were expected to have the same pollen donor parent. Moreover, the effective number of male parents per progeny arrays was 7.86 among the ten studied populations (*N*_ep_ = 7.86), which was lower than that of native populations in Mexica (*N*_ep_ = 8.97)^30^.

There were no significant relationships between multilocus outcrossing rates and population size, population density, altitude, latitude or longitude (*P* > 0.05). However, the smallest population YQ (population size = 60) had a high outcrossing rate (0.783 ± 0.07). There was no evidence of a relationship between biparental inbreeding and population size (*P* > 0.05), but relatively high level of biparental inbreeding was found in two small populations (WSL, 0.137 ± 0.023; YQ, 0.112 ± 0.029).

### Genetic diversity

The ten invasive populations of *S. rostratum* exhibited low to intermediate genetic diversity in China. Table 2 shows a summary of genetic diversity parameters in the ten studied populations. The expected heterozygosity (*H*_e_) ranged from 0.049 to 0.415; and the polymorphism information content (PIC) varied from 0.043 to 0.357. In the ten Chinese populations, the YG population had the lowest average number of alleles per locus, polymorphism information content, expected heterozygosity and observed heterozygosity (N_a_ = 1.45; PIC = 0.043; *H*_*e*_ = 0.049; *H*_*o*_ = 0.036); the WSL population had the highest polymorphism information content, expected heterozygosity and observed heterozygosity (PIC = 0.357; *H*_*e*_ = 0.415; *H*_*o*_ = 0.304). We detected more homozygosity than expected across loci within populations, as the inbreeding coefficient (*F*_is_) was positive in all populations (varying from 0.166 in population CY to 0.313 in population NZ). And Chinese populations’ genetic differentiation was high (*F*_st_ = 0.216).

**Table 2:** Genetic diversity estimates and exclusion probabilities for sampled populations of *Solanum rostratum* in China.

Population	N_ind_	P	N_a_ (range)	PIC	PE_sp_	*H*_e_ (SE)	*H*_o_ (SE)	*F*_is_
BC	144	11	1.45 (1–2)	0.138	0.656	0.174 (0.020)	0.123 (0.015)	0.294^**^
CJ	144	11	2.00 (1–4)	0.240	0.459	0.287 (0.022)	0.173 (0.016)	0.397^**^
CY	144	11	3.18 (2–7)	0.310	0.341	0.355 (0.020)	0.296 (0.018)	0.166^**^
YG	144	11	1.45 (1–3)	0.043	0.924	0.049 (0.010)	0.036 (0.008)	0.267^**^
WSL	144	11	3.00 (1–7)	0.357	0.195	0.415 (0.023)	0.304 (0.020)	0.269^**^
NZ	144	11	2.82 (1–4)	0.140	0.757	0.161 (0.015)	0.110 (0.010)	0.313^**^
SLZ	139	11	4.45 (2–8)	0.305	0.272	0.357 (0.025)	0.284 (0.022)	0.207^**^
MY	144	11	2.73 (1–7)	0.257	0.379	0.302 (0.023)	0.244 (0.020)	0.192^**^
YQ	144	11	2.36 (1–5)	0.223	0.507	0.270 (0.020)	0.209 (0.017)	0.225^**^
TZ	144	11	2.82 (1–6)	0.306	0.317	0.354 (0.023)	0.256 (0.017)	0.277^**^
Species level	1435	11	7.55 (4–16)	0.482	0.053	0.545 (0.018)	0.201 (0.011)	0.225^**^

Note: N_ind_ = number of individuals successfully genotyped; P = number of polymorphic loci; N_a_ = average number of alleles per locus; PIC = polymorphic information content; PE_sp_ = combined probability of exclusion of a single parent; *H*_e_ = expected heterozygosity; *H*_o_ = observed heterozygosity; *F*_is_ = inbreeding coefficient calculated using FSTAT, and associated *P*-values were determined using 11000 randomizations, and a nominal level for multiple tests of 1/1000. ^**^ *P*-value < 0.01.

Chinese populations showed relatively lower genetic diversity (Table 2) than Mexican populations. As the polymorphism information content (PIC = 0.482), expected heterozygosity (*H*_e_ = 0.545) and observed heterozygosity (*H*_o_ = 0.201) were lower than those of the Mexican populations (PIC = 0.532; *H*_e_ = 0.579; *H*_o_ = 0.368)^30^. The inbreeding coefficient of Chinese populations (*F*_is_ = 0.225) was lower than Mexican populations (*F*_is_ = 0.256)^30^. While Chinese populations (*F*_st_ = 0.216) had higher genetic differentiation than Mexican populations (*F*_st_ = 0.159)^24^.

## Discussion

Evaluating the mating system of invasive species is an important task to better understand the conditions in which self- or cross-fertilization or a mixed mating strategy is favored in invasive populations^1,20^. In many cases, we expect an evolutionary shift towards a higher frequency of self-fertilization, because selfing provides reproductive assurance under the initial founder event, resulting in Allee Effects. *Solanum rostratum* is a self-compatible annual herb that relies on insect visitation for cross-pollination. Based on our field experiments, in which we excluded pollinators using mesh bags, the capacity for self-fertilization in *S. rostratum* is very low in North China. Our estimates of mating patterns in *S. rostratum* indicated a mixed mating system dominated by outcrossing, and the mating system of *S. rostratum* did not experience an evolutionary transition during its invasion of China. Recent analyses of the accumulated data have highlighted the high frequency of species with mixed mating systems^17,31^. However, the evolution of mixed mating strategies of invasive species is complex. On one hand, selfing can produce seeds under conditions of low density or during frequent episodes of colonization with severe inbreeding depression. On the other hand, outcrossing can maintain high genetic diversity within populations. There may be a balance between selfing and outcrossing for *S. rostratum* under different conditions, and efforts to explore the reproductive success of different populations can help us better understand the maintenance of mixed mating systems of *S. rostratum* during invasion.

There are many ecological factors that can influence outcrossing rates. Variation in flower design and display has been shown to influence pollinator visitation rates^32,33^, and the rates of pollinator visitation can directly affect the amount of outcrossing rates in self-compatible plants^19,20^. The flowers of *S. rostratum* exhibit monomorphic enantiostyly and strong herkogamy. In addition, it has been hypothesized that the high outcrossing rate in this species is probably facilitated by its floral morphology^29,30^. Moreover, *S. rostratum* is a buzz-pollinated plant, and insect size is the main determinant of whether a visitor acts as a pollinator or a pollen thief^34^. During the visit of effective pollinators, heteranthery results in the deposition of pollen on one side of pollinator’s body against the style. Pollen from one floral morph will be deposited on the stigma of the opposite floral morph, thus reducing pollinator-assisted selfing within flowers. However, geitonogamy is unavoidable in large individuals, in which left- and right-styled flowers occur at the same time.

The mating system of flowering plants is influenced not only by its flower design and display but also by population size, geographic location and habitat fragmentation^20,35^. In our preliminary study, in which we performed pollen addition experiments, we reported that pollen addition significantly increases fruit set and seed production and that smaller populations suffer from more severe pollen limitation^36^. The ecological context of pollen limitation can negatively impact plant-pollinator interactions and generate diverse selective forces on plant mating systems. In this study, we did not find a significant relationship between multilocus outcrossing rates and population size, altitude, latitude or longitude. However, the smallest population, YQ, had a high outcrossing rate (0.783 ± 0.07), whereas the largest population, CJ, had a relatively low outcrossing rate (0.543 ± 0.069). There is evidence that the outcrossing rate was associated with the floral display^17^. In our field investigations, plants in the YQ population were small; some individuals only opened one or two flowers per day, and the flowering season lasted for more than four months, which may reduce pollen transfer between the same plants. By contrast, plants in the CJ population were relatively large; and most individuals opened more than five flowers per day during the flowering season, which may promote geitonogamous selfing. Therefore, the floral display may have an impact on the outcrossing rate in this species; however, this phenomenon needs further study.

In this study, population CY had the highest outcrossing rate (0.834 ± 0.064) in the ten studied populations in China, and population CY also has the highest outcrossing rate in the world based on the present studies. There are reasons explaining why the highest outcrossing rate occurred in population CY. First, population CY has a relatively long invasion history, and it is the first introduced population reported in China^24^. This may have given the species enough time to adapt to the environment and co-evolve with local pollinators. Second, population CY is located in riversides without habitat fragmentation, which provides relatively favorable conditions for *S. rostratum*. Third, the genetic diversity of population CY is very high (PIC = 0.310), which may guarantee the ability to maintain high outcrossing rates^1,4,13^. By contrast, population YG, which had the lowest outcrossing rate (0.492 ± 0.225), is the last population that we found in this study and is located on the side of a railway. The genetic diversity of population YG is also the lowest (PIC = 0.043). The difference in the two populations may indicate that the invasion history, living habitat and genetic background could influence the mating system of *S. rostratum*. And further studies should be conducted to explain the maintenance of mixed mating system in *S. rostratum*.

Moreover, the offspring used for mating system analysis were seedlings, and this may overestimate the outcrossing rate of *S. rostratum*. The mean germination rate of the ten populations was high (0.94 ± 0.05), and the germination rate was not correlated with the outcrossing rate. However, in many outcrossing species, the germination rate of self-fertilized seeds is lower than that of outcrossed seeds because of inbreeding depression^37^. Therefore, inbreeding depression functioning in the offspring of *S. rostratum* may lead to a higher estimate of the outcrossing rate. Further studies to determine the extent of inbreeding depression in *S. rostratum* will provide additional reproductive information on this highly outcrossing species.

In conclusion, our results suggest that a mixed mating system dominated by outcrossing of *S. rostratum* occurs in its invasive Asian range. The average multilocus outcrossing rate was 0.69 ± 0.12 (ranging from 0.49 to 0.83), with an average effective number of 7.86 male parents in Chinese populations. Compared with the high level of outcrossing rates (0.70 ± 0.03) in native populations in Mexico^30^, these results indicate that there was no evolutionary transition from outcrossing to selfing during *S. rostratum*’s invasion of China. Moreover, there were no significant differences between the outcrossing rates and population size, altitude, latitude or longitude. We speculate that high outcrossing rates in invasive populations of *S. rostratum* may be facilitated by precise pollination owing to herkogamy and monomorphic enantiostyly.

## Methods

### Sampling design

**Figure 3:**
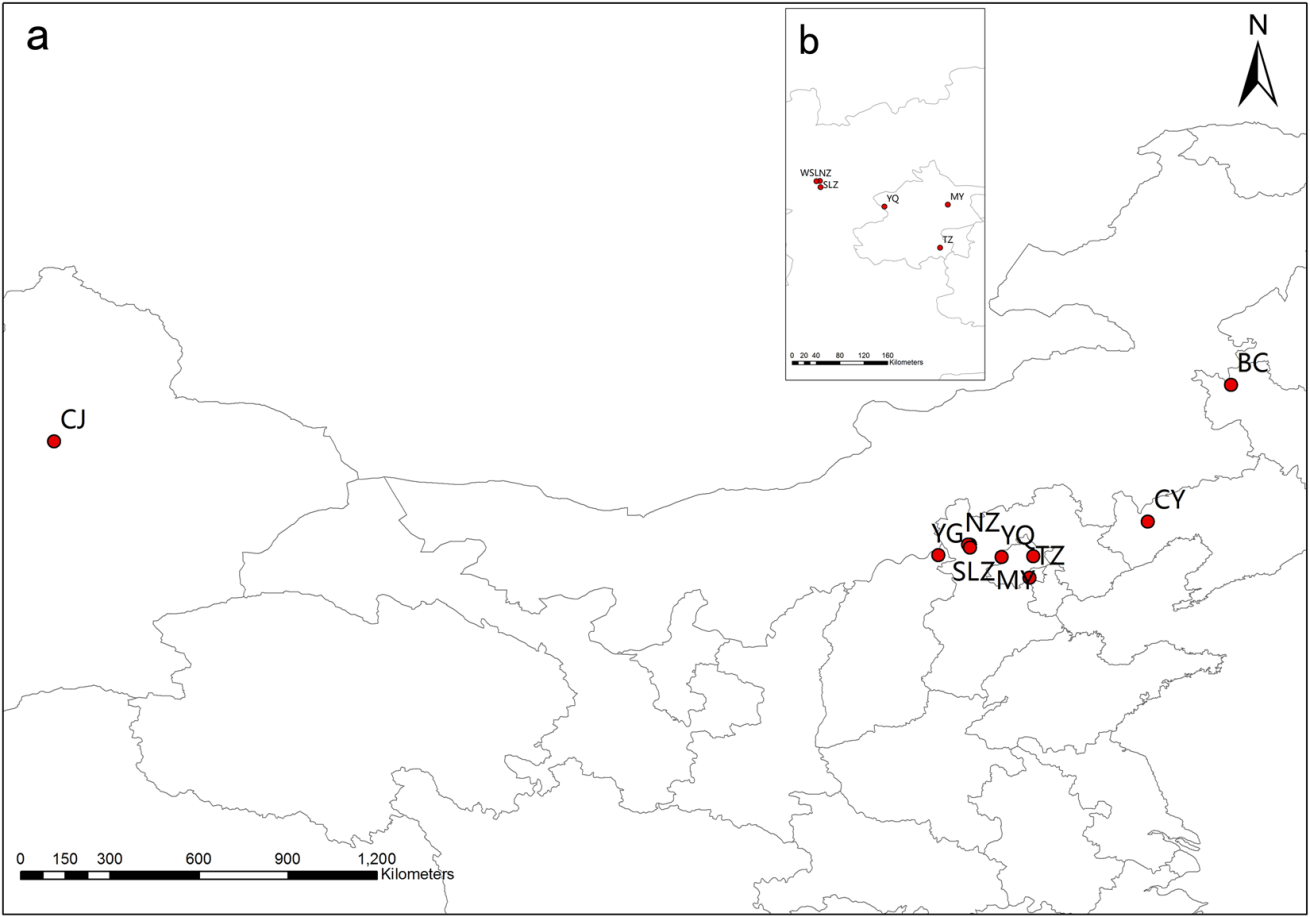
Geographical distribution of ten populations of *Solanum rostratum* sampled from North China. (**a**): ten Chinese sampling populations of *Solanum rostratum*, the dot between YQ and YG represents there populations located in Hebei province (WSL, NZ, SLZ). (**b**): details with enlarged scale for the six populations located in Hebei province (WSL, NZ, SLZ) and Beijing (MY, YQ, TZ). Population names are provided in Table 1. This figure was produced using ArcGIS software version 10.1 (http://www.esrichina.com.cn/softwareproduct/ArcGIS/).

To estimate mating parameters in *S. rostratum*, ten Chinese populations were sampled in a variety of habitats. The ten populations cover a large area of the invasive distribution in China and exhibit a wide range of population size (Table 3; Fig. 3). We randomly collected fruits from 30 individuals per population in October and November 2010. We then extracted seeds from the fruits and placed them in airtight containers at 4 °C.

**Table 3:** Locations and sample size of ten Chinese populations of *Solanum rostratum* used for mating system study.

Population	Latitude (N)	Longitude (E)	Elevation (m)	Population size	Population density (Ind. ·m^−2^)	Genotyped offspring (Maternal families)	Germination rate	Habitat
BC	45° 35′23.4″	122° 50′11.3″	154	2000	0.65	144 (12)	0.8992	Railway side
CJ	43° 52′35.8″	87° 09′50.0″	790	5000	0.53	144 (12)	0.9625	River side
CY	41° 27′28.4″	120° 18′58.4″	190	500	1.90	144 (12)	0.9708	River side
YG	40° 26′1.8″	113° 58′14.2″	1048	300	0.21	144 (12)	0.9792	Railway side
WSL	40° 45′38.1″	114° 55′16.8″	729	250	1.35	144 (12)	0.9333	Road side
NZ	40° 45′18.1″	114° 52′1.5″	711	300	0.59	144 (12)	0.9247	Railway side
SLZ	40° 39′57.5″	114° 55′47.7″	650	120	0.56	139 (12)	0.9917	Railway side
MY	40° 24′10.8″	116° 50′48.0″	85	3000	0.27	144 (12)	0.8375	River side
YQ	40° 22′26.7″	115° 53′29.0″	514	60	2.40	144 (12)	0.9333	Road side
TZ	39° 45′12.2″	116° 43′49.9″	17	1500	2.16	144 (12)	0.9386	River side

For each population, we detected the genotypes of all seedlings randomly sampled from 12 mother plants and 12 young seedlings per mother plant. We did not use seeds because they were rich in endosperm, and DNA extraction yields poor-quality material^30^. To maximize the germination rate, the seeds were pretreated with a 1000-ppm solution of gibberellic acid for 24 h. We sowed 50 seeds per family (5 seeds per fruit and 10 fruits per family) in plastic seedling-raising dishes in March 2011 and placed the dishes in a greenhouse at Beijing Normal University. Finally, we randomly sampled a total of 1435 seedlings belonging to 120 maternal families (Table 3).

### DNA extraction and genotyping

We extracted genomic DNA from leaves dried in silica gel of the 1435 offspring using a plant genomic DNA kit (Tiangen, Beijing, China). We scored the genotypes of the DNA samples using 11 pairs of microsatellite primers, which were previously developed for *S. rostratum* ^38^. The 11 loci were amplified using multiple polymerase chain reactions (PCR) with a Microsatellite PCR kit (Qiagen, Shanghai, China). The PCR amplifications were performed using a Bio-Rad thermocycler as follows: one cycle of 95 °C for 5 min; 30 cycles of 95 °C for 30 s, 58 °C for 180 s, and 72 °C for 30 s; and a final extension step at 60 °C for 30 min^38^. The PCR products were sequenced using an ABI 3730xl capillary sequencer with GeneScan 500 LIZ as the internal size standard. The results were analyzed with GeneMapper Software version 3.7 (Applied Biosystems, Foster City, CA, USA).

### Data analysis

We analyzed the number of alleles (N_a_), expected heterozygosity (*H*_e_), observed heterozygosity (*H*_o_), polymorphism information content (PIC) and the combined probability of exclusion of a single parent (PE_sp_) for each population using the computer programs GENALEX version 6.4^39^ and CERVUS version 3.0^40,41^. We estimated the inbreeding coefficient (*F*_is_) using FSTAT version 2.9.3^42^. The statistical significance of *F*_is_ was tested using 11000 randomizations, and the threshold was adjusted for multiple comparisons using the Bonferroni correction. To test differences in genetic variation between the ten populations in China, we estimated the population differentiation (*F*_st_) among populations using 1000 permutations in FSTAT version 2.9.3^42^. The presence of null alleles was calculated using Micro-Checker software^43^.

Mating system parameters for each population were estimated using the expectation-maximization method of the computer program MLTR version 3.2^44^. We estimated the multilocus outcrossing rate (*t*_m_), single-locus outcrossing rate (*t*_s_), and outcrossing rate between related individuals (*t*_m_−*t*_s_). The outcrossing rate between related individuals (*t*_m_−*t*_s_) can be used to estimate biparental inbreeding. We also calculated the correlation of outcrossing rate (*r*_t_), the correlation of paternity within sibships for both multilocus cases and single cases (*r*_pm_ and *r*_ps_, respectively), and the extent of outcrossed paternity by related male parents (*r*_ps_−*r*_pm_). The correlations of paternity (*r*_pm_ and *r*_ps_) represent the fraction of siblings that share the same male parents^44^. We calculated the effective number of male parents per progeny arrays among the ten populations as *N*_ep_ = 1/${\overline{{\rm{r}}}}_{{\rm{pm}}}$ ^45^. Standard deviations were computed based on 1000 bootstrap values, with the resampling of entire maternal families. For tests of statistical significance, we examined the distribution of 1000 bootstrap values to determine whether outcrossing rates differed significantly from 1.0^46,47^. When values were significantly less than 1.0, the species exhibited a mixed mating system. Comparison of outcrossing rates between Chinese populations and Mexican populations was done with independent samples t-test^11^. The relationships between multilocus estimates of outcrossing rate and population size, altitude, latitude and longitude were analyzed by Spearman rank correlation tests. In addition, the relationship between biparental inbreeding and population size was also analyzed by Spearman rank correlation tests.
